# Influence of Immune Responses in Gene/Stem Cell Therapies for Muscular Dystrophies

**DOI:** 10.1155/2014/818107

**Published:** 2014-05-19

**Authors:** Andrea Farini, Clementina Sitzia, Silvia Erratico, Mirella Meregalli, Yvan Torrente

**Affiliations:** ^1^Laboratorio Cellule Staminali, Dipartimento di Fisiopatologia Medico-Chirurgica e dei Trapianti, Università degli Studi di Milano, Fondazione IRCCS Ca' Granda Ospedale Maggiore Policlinico, Centro Dino Ferrari, Via F. Sforza 35, 20122 Milano, Italy; ^2^Ystem s.r.l., Via Podgora 7, 20122 Milano, Italy

## Abstract

Muscular dystrophies (MDs) are a heterogeneous group of diseases, caused by mutations in different components of sarcolemma, extracellular matrix, or enzymes. Inflammation and innate or adaptive immune response activation are prominent features of MDs. Various therapies under development are directed toward rescuing the dystrophic muscle damage using gene transfer or cell therapy. Here we discussed current knowledge about involvement of immune system responses to experimental therapies in MDs.

## 1. Introduction

The involvement of inflammation in muscular dystrophies (MDs) has been known for years. However, molecular mechanisms underlying immune system activation are not completely understood. Inflammation and innate immune response activation are firstly a consequence of physiological function of skeletal muscle, but their chronic activation is determined by continuous cycles of muscle fibers degeneration/regeneration. MDs are a heterogeneous group of diseases caused by mutations in different components of sarcolemma, extracellular matrix, or enzymes [1]. Despite differences in genetic background and symptoms, MDs share some characteristic features such as progressive muscular wasting, fibrosis and atrophy, and various degrees of inflammatory infiltrates. Here we described the well-known involvement of the cells of the immune system in the development of the pathological signs of the most frequent forms of MDs—Duchenne Muscular Dystrophy (DMD) and dysferlinopathies (LGMD2B)—and the emergent role of these cells in the facioscapulohumeral muscular dystrophy (FSHD). Moreover, we investigated the relationship between immune system and gene or cell therapy in the treatment of these diseases. DMD is characterized by mutations in dystrophin gene: its absence at the sarcolemma reduces the stability of plasmamembrane and renders muscular fibers more prone to contraction-induced injury [1]. In LGMD2B the mechanism of membrane repair is inefficient due to the absence of dysferlin protein, which probably regulates vescicular trafficking [2]. Molecular mechanisms underlying FSHD are not fully understood but it is known that the contraction of a repeated region in chromosome 4q35 leads to toxic activation of DUX4 gene (i.e., normally silenced), which probably acts like a transcription factor [3]. As we discussed below, a certain degree of inflammation is always present in whatever form of MD, so that this condition is probably due to the muscular degeneration itself. However some aspects, such as complement system deposition or specific lymphocytes activation, are typical of one form of MD suggesting a correlation with the genetic background. Finally we discussed how immune system activation could affect gene or cell therapy and how it could be the target of new treatments.

## 2. Immune System Activation in Skeletal Muscle

In physiological condition, skeletal muscle contains resident immune cells, mainly macrophages, that exert multiple roles such as phagocytosis of cellular debris and microbes, secretion of cytokines and growth factors, antigen-presentation. Conversely, following pathophysiological stimuli, skeletal muscle is invaded by several immune cells that secrete soluble molecules, affecting the viability and transcriptional activities of regenerative muscle cells. Unfortunately, the complex mechanisms that regulate the interplay among immune system cells and skeletal muscle stem cells and their modulation of muscular regeneration are far from being really understood. In particular innate immunoresponse of the muscle to injury is mediated by Th1 cytokines (that are the cytokines expressed by a particular subset of T helper cells named Th1) which triggers the activation of classic M1 proinflammatory macrophages, which in turn promote the production of prostaglandins, cytokines, and chemokines [4]. Following the early invasion of macrophages/neutrophils, tumor necrosis factor alpha (TNF-*α*) is highly expressed, activating macrophages to the M1 phenotype, and also inducing the production of other proinflammatory cytokines. Among them, the activation of nuclear factor kappa-light-chain-enhancer of activated B cells (NF-kB) increases proliferation and inhibits differentiation of muscle stem cells. In fact, NF-kB allows the expression of transcripts needed for cell cycle progression and causes destabilization of MyoD mRNA and degradation of MyoD protein, negatively affecting the capacity of muscle to terminal differentiation [5]. Furthermore, recent findings demonstrated that the secretion of the TNF-*α* in the injury site is necessary for the attraction of satellite cells and, thus, for the promotion of muscle regeneration [6]. In a second time, as M1 macrophages reached the peak of concentration in injured/regenerative muscle, Th2 cytokines (IL-4, IL-10, and IL-13) stimulation promotes a switch toward M2 anti-inflammatory macrophages, which diminish the inflammatory response and promote tissue repair [7, 8]. The transition from a Th1 inflammatory response to a Th2 inflammatory response is closely correlated with a transition from the early proliferative stage of myogenesis (driven by the transcription factors Myod and myf-5) to the terminal stages of myogenesis (driven by Myogenin and MEF2). Interestingly, the functional linkage between M1/M2 differentiation and myogenic compartment was suggested, as the disruption of the Th1 to Th2 transition causes the failure of the transition from proliferative to differentiation stages of myogenesis, in particular at a stage at which satellite cells are activated to proliferate and express MyoD [6]. Similarly, different works demonstrated the fundamental role of M2 macrophages in promoting muscle regeneration, as the depletion of this subpopulation of macrophages prevented increases in muscle fiber diameter and diminished the ability of muscles to repair, to differentiate, and to regenerate [9]. Muscular alterations render the myofibers more vulnerable to contraction-induced injury so that continuous activation of the immune system is present. Chronic inflammation ultimately ends in fibrosis deposition and atrophy, a process mainly mediated by a transition from M2a macrophages to M2c macrophages [10–12]. Contemporary macrophages and myokines secreted by muscle fibres recruit additional immune cells, including T cells, which exacerbate muscular damage. In DMD muscles macrophages are the most abundant immune cells, but T cells, B cells, and dendritic cells (DCs) are also present. Infiltrating T cells are predominantly CD4+, and smaller numbers of cytotoxic CD8+ T cells were observed [13]. In addition, specific T cell receptor gene rearrangements were observed in clonally activated T cells, within dystrophic muscle fibers [14]. Furthermore, recently, Mendell et al. observed specific autoreactive T lymphocytes, directed against dystrophin epitopes, in blood of dystrophic patients (DMD) [15]. Several authors demonstrated that the ablation of T (or T/B cell) cells ameliorated muscular pathology in dystrophic animal models [16–18]. This amelioration was due both to direct ablation of cytotoxic T cells and to reduced secretion of proinflammatory cytokines. Among them, higher levels of TNF-*α*, IL17, IL6, and TGF-*β* were observed in DMD muscles than in healthy ones [19]. In particular, TNF*α* has been suggested to promote necrosis through a regulated process involving kinase activity of receptor-interacting protein 1 (RIP1) [20]. More recently, Burzyn and colleagues described a population of regulatory T (Treg) cells expressing Foxp3 and CD4 that rapidly accumulated in the acutely injured skeletal muscle of mice, similar to myeloid cells. They demonstrated that these cells exerted a fundamental role in regulating muscle repair by the expression of specific growth factors [21]. Immune system activation is not a predominant feature only of DMD; indeed other forms of MDs share the same characteristic, even if different subpopulations of immune cells might be affected. Inflammatory cells were detected in both MM and LGMD2B patients around necrotic fibers. Dysferlin deficiency reduced the capacity of muscle membranes to repair after injury prolonging the recruitment and the activation of inflammatory cells. Enhanced phagocytic activity of dysferlin-deficient macrophages was reported [22] together with components of i(NALP)-3 inflammasome pathway upregulated and activated [23]. Specific deposition of membrane-attack complex (MAC) at the surface of muscle fibers was observed in dysferlin-null mice, demonstrating an important role of the complement factors in exacerbate muscular damage [24]. Skeletal muscle under physiological condition do not constitutively express major histocompatibility complex (MHC) class I molecules: different stimuli however can induce their expression, such as the proinflammatory cytokines IFN-*γ* and TNF-*α* [25]. Expression of MHC class I on the surface of muscle cells is an early feature in human idiopathic inflammatory myopathies, also preceding inflammatory infiltrates [26]. It was demonstrated that human skeletal muscle myoblasts, once stimulated to express MHC class I or even class II, can effectively present antigens to autologous, antigen-specific CD4+ T cells [27]. In dysferlinopathies abnormal MHC-I expression was observed in degenerating/regenerating fibers usually closed to inflammatory cluster cells. A direct pathogenic role for immunity has never been demonstrated in FSHD. Inflammatory features were evident in biopsies from FSHD patients [28, 29], whereas a pilot trial involving prednisone did not improve strength in FSHD muscles [30]. More recently, magnetic resonance imaging (MRI) was used as powerful method to identify specific areas of the muscles in different disorders [31]. This way, Tasca et al. demonstrated a correlation between hyperintensity in T2-STIR sequences and histological muscle abnormalities and showed that these areas were characterized by inflammation [32]. Moreover, to better elucidate the possible pathological mechanisms underlying inflammatory and immunological processes, they studied the amount of activated immune cells in the blood of FSHD patients and their expression of cytokines and other immune regulators. They found a significant amount of CD8+ T-cells in the endomysium of FSHD muscles, close to nonnecrotic fibers. They also noted that FSHD patients overexpressed pSTAT1 (regulator of Th1 cells), pSTAT3 (regulator of Th17 cells), and t-bet (regulator of both innate and adaptive immunity) that cause overproduction of IL12/IL23p40, IFN-*γ*, TNF-*α*, IL6, and IL10 related to controls [33]. The excessive secretion of IL6 driven by monocytes probably caused CD8+ T cell activation, allowing uncontrolled proliferation and effector functions. Interestingly, they did not assess any modification of the complement system as suggested in the dysferlinopathies [34]. A study published in 2012 confirmed that in the blood of FSHD patients several genes that mediated innate and adaptive immune response were upregulated, assessing that inflammation had a central role in the development of pathological phenotype [32].

## 3. Inflammation-Based Therapeutic Strategies

Despite all efforts exerted, steroids are the only current treatment available for DMD. The exact mechanism of action of steroids in DMD patient is not fully characterized but the beneficial effects observed are probably due to their immunosuppressor ability. For example, it is thought that glucocorticoids promote a shift from M1 to M2 anti-inflammatory macrophages, as observed in patients treated with prednisone (0.75 mg/kg/day) during 6 months [35]. As we discussed above, steroids treatments are also associated with lower levels of autoreactive T-lymphocytes in DMD patients [36]. The involvement of cytokines and other inflammatory actors in MDs opened new therapeutic strategies. In particular, modulation of proinflammatory cytokines, such as TNF*α*, is currently under investigations. Infliximab and Etanercept (TNF*α* neutralizing antibody and soluble TNF*α* receptor, resp.) both showed reduction in fibrosis deposition and in myofiber necrosis in mdx mice [37, 38]. Similarly, blocking chemokine-mediated signalling, through chemokine-receptor antibodies (including an anti-CCR2) seemed to be promising. Major concerns with this strategy involved the abundance of chemokines that are upregulated in DMD patients and the interpatient variability. Furthermore, as we discussed above, chemokines are necessary to attract noncytotoxic macrophage subpopulations, which are responsible for myofibers regeneration [9]. In the same way TGF-*β* blocker suramin and the TGF-*β*1 antagonist pirfenidone decreased fibrosis in mdx mice [39, 40]. Immunomodulatory strategies have been investigated in other forms of MDs such as dysferlinopathies. Unfortunately, anti-inflammatory glucocorticoids gave negative results in treated patients probably because of side effects [41, 42]. However, alternative strategies, such as intravenous immunoglobulins and Rituximab administration, showed partial benefits in LGMD2B patients although B lymphocytes are not a major component of infiltrates in this disease [43]. Similar to what observed in DMD patients, the administration of Etanercept resulted in dose-dependent reductions of inflammation, necrosis, and fatty/fibrous change in dysferlinopathic SJL/J mice [44]. Recently, Halofuginone, a coccidiostat (T helper 17 cells inhibitor), was shown to significantly improve dystrophic features in a dysferlin-deficient mouse model [45]. As discussed above, complement system plays a central role in promoting myofiber necrosis in dysferlinopathies, so that it represents an attractive target for therapies. In this sense genetic ablation of C3 improved muscle phenotype in dysferlin-deficient mice [24]. To date, immunomodulatory therapies for FSHD, including a prednisone pilot trial, failed to demonstrate beneficial effects [30].

## 4. Influence of Immune Responses in Gene Therapy

Gene therapy is commonly used to correct or to replace genes whose mutations cause fatal disease. It is characterized by 3 critical steps: the genes that are transferred, the target tissue, and the carrier of the gene. The carrier has to allow the entry of the gene into damaged tissues: retrovirus, lentivirus, adenovirus, and adenoassociated virus (AAV) are commonly used as vectors. AAVs can infect long-lived postmitotic cell; their DNA can be integrated into the host cell's genome and donated gene is long-term expressed [46]. As the administration of such viral vectors could cause host immune response, clinical protocols for gene therapy require that sustained therapeutic levels of the transgene are achieved, with no apparent vector-related toxicities in the patient [47, 48]. In this section, we will describe the effects of administration of AAVs in the most common forms of neuromuscular disorders.

### 4.1. Adeno Associated Virus (AAVs) and Immune Reactivity

AAVs are nonpathogenic and replication-defective—as they did not possess any viral protein ORFs—that can infect nondividing cells. To date, 12 different AAV serotypes (AAV1-12) are utilized in gene therapy approaches, regarding liver (AAV8), cardiac, and musculoskeletal tissues (AAV1, 6 and 9), central nervous system (AAV5 and 9), and eye (AAV4 and 8) [47]. AAVs have low immunogenicity compared to adenovirus as they did not transduce efficiently macrophages, mature DCs, and other antigen-presenting cells (APCs); however, different clinical trials demonstrated that these vectors can cause immunological responses [49]. Furthermore, it is known that AAVs are nonintegrating vectors but insertional mutagenesis could occur [50]. Zaiss and Muruve showed that APCs can take up exogenous AAV antigenic peptides by endocytosis; consequently, AAVs are released in the endosomes, entering into the nucleus and viral genome induces uncontrolled gene expression [51]. This way, innate immune signalling pathways are triggered and the secretion of several factors that regulate inflammatory response allows the expression of proinflammatory cytokines and chemokines [52, 53]. All these molecules favour the infiltration of neutrophils, macrophages, and DCs that kill the transduced cells directly or initiate a more specific T- and B-cell response [54]. Alternatively, it was demonstrated that the complement system—a component of the innate immune system that promotes inflammation [55]—had an important role in the host response to AAV vectors [56], as their interaction caused the activation of macrophages against AAVs [56]. Other than innate immunity, AAVs could be identified and destroyed by the adaptive immune responses. Once the vector is brought inside the host cells, the viral capsid is degraded so that antigenic peptides are cross-presented to MHC I molecules and CD8+ T cells destroy the transduced host cell [57]. Activated CD8+ T cells can elicit indirectly the function of CD4+ T cells by secreting proinflammatory cytokines (IFN*γ*, TNF-*α*, and IL-2) [57]. Alternatively, APCs could present the AAVs to T-cell receptor (TCR) of CD4+ T cells by means of MHCII complex [58]; this way, CD4+ T cells allow the expression of proinflammatory cytokines (IL-2, IL-4, and IL-5) that can activate B cells [59]. At this point, specific antibodies against the particles of AAVs are produced and then eliminated by neutrophils and DCs [59]. As eyes are considered ideal organs for AAVs infusion for their immune-privileged status, the works of Maguire et al. [60] and Hauswirth et al. [61] demonstrated that the ocular injections of AAV vector are a safe and efficacious technique to ameliorate visual function in LCA patients. For all the other organs, immunosuppression protocols were proposed to reduce or prevent host immune response following AAVs injection. In the case of degenerative disorders such as haemophilia and muscular dystrophies, continuous administration of AAVs is required for therapeutic purposes. Unfortunately, secondary exposure to the vector strengthens the risk of activation of memory T- and B-cells, immune reactivity events, and vector elimination. It was suggested that these problems could be overcome with short-term immune suppression treatment in the initial phase of vector infusion [62, 63]. In addition to immunological questions, the other milestone of gene therapy is its translation into large animals rather than mice. To this end, Valentine et al. used the beagle-based canine X-linked muscular dystrophy (CXMD) which shared with DMD patients several pathological features [64]. Following injection of rAAV2 driven by muscle-specific promoter, they described a significant infiltration of cells 2 weeks after the treatment. In particular they identified CD4+ or CD8+ T lymphocytes in the interstitial spaces of the injected muscle while CD11b+ cells and B cells were found in clusters among infiltrating cells. More interestingly, muscle fibers upregulated MHC classes I and II molecules [65]. Experiments of administration of the rAAV expressing no transgene into the CXMD muscles suggested that the strong immune response that takes place in treated muscles and that causes the elimination of transduced myofibers is due to transgene product and not to AAV capsid [65].

### 4.2. AAV Treatment in Neuromuscular Disorders

Adriouch et al. described a strategy to inhibit the undesirable immune activation that follows muscle gene transfer. The cytotoxic T-Lymphocyte Antigen 4 (CTLA-4 or CD152)—a protein receptor that downregulates the immune system that is present on the surface of T cells, leading the cellular immune attack on antigens—was used to block the costimulatory signals that are required early during immune priming. In combination with the supplementation of programmed cell death protein 1 (PD-1) to inhibit T cell functions at the tissue sites, they efficiently modulated the immune response of AAVs transduced muscle cells [66]. Similarly, Lorain et al. characterized the immune response to the AAV1 vector in the DMD murine model, the mdx mouse. They explored methods to block interactions between T cells, antigen-presenting cells and B-cells by interfering with the costimulatory pathways B7/CD28 (CTLA4) and CD40L/CD40, using two specific agents, CTLA4/Fc and MR1. They demonstrated that this immunomodulatory treatment completely blocked the formation of antibodies against the AAV1 vector and enhanced the expression of dystrophin in several muscles [67]. Mendell and coworkers showed that LMGD patients injected with AAV1 had both humoral and cellular immunity against the capsid even if the expression of the transgene was not impaired by the immune response [68]. To avoid the recognition of AAVs by T cells and to ameliorate the efficiency of transduction, the immunogenic epitopes of the capsid were masked. Furthermore, tissue-specific promoters were used to modulate the expression of the transgene only in the target tissues, avoiding the recognition by APCs and subsequent activation of the immune system [54].

#### 4.2.1. Gene Therapy and DMD

According to their low immunogenicity coupled with their “cargo” capacity, AAVs were largely used to treat fatal neuromuscular disorders. Although the genome of AAV persists into muscle for several years, it was demonstrated that the integration of the AAV genome into host myogenic cells was largely inefficient and disappeared rapidly with time. Schnepp et al. suggested that very little rAAV vector DNA integrated in transduced mouse muscle and that viral DNA persisted only as concatameric episomes [69]. Despite these problems, different serotypes of AAV were used with encouraging results in model mice of DMD. rAAV serotypes 6 and 8 efficiently delivered the microdystrophin cassette to skeletal muscle in the mdx mouse through the vasculature [70]. Moreover, they achieved skeletal muscle transduction also in compartments far from the site of injection, such as limb muscles [70]. Similarly, Wang et al. demonstrated that AAV8 is the most efficient vector for crossing the blood vessel barrier and AAV8-mediated gene expression persisted in muscle and heart, but diminished in tissues undergoing rapid cell division [71]. As direct injection of rAAV1 was efficient and safe [69], this vector was transplanted into femoral artery of mdx mice, allowing robustly reexpression of dystrophin [72]. However, it was showed that the capacity of transduction of rAAV6 and 8 was significantly better than rAAV1, concerning the amelioration of isometric force in treated mice [73]. Another important problem in the treatment of DMD patients arose for their possible immune response to vectors-carried dystrophin. The risk of T-cell immunity can be strongly reduced whether the differences between the defective self-gene and the therapeutic transgene are limited. It could be the case of pathologies that are provoked by a small number of missense mutations but the pathologies that are caused by large genomic deletion of specific genes (such as dystrophin in DMD and BMD) increased the risk of this kind of immunological reaction. In fact the protein in DMD/BMD patients is absent or present in abnormal form so that gene transfer mediated by viral vectors could cause the development of immune responses to previously unseen epitopes. As a confirmation, antibodies specific to the donor dystrophin were seen in BMD and DMD patients that received, respectively, cardiac [74] and donor myoblast transplantation [75, 76]. To avoid that other proteins introduced with dystrophin in these experiments could alter the specific immune response to dystrophin, Ferrer et al. injected naked plasmid DNA into skeletal muscle of mdx mice to be sure that no potential neoantigens were introduced and described a specific immune responses to dystrophin [77]. Although the first DMD trials based on myoblast transplantation showed a partial expression of dystrophin [76, 78, 79], they failed probably for immunological reaction of these patients to dystrophin. Ferrer et al. demonstrated that, following injection into mdx mice of plasmid encoding for minidystrophin, the newly formed dystrophin positive myofibers were destructed four week after the treatment through a cell-mediated immune response. In particular, cytotoxic CD8+ T cells were identified in clusters around the dystrophin + fibres in the injected leg [77]. Interestingly, they also noted that full-length dystrophin was less immunogenic than the minidystrophin, probably for the presence of suppressor epitopes in the rod domain that is deleted in the minidystrophin [77]. Similarly, Yuasa et al. treated mdx mice modified to express minidystrophin with AAVs; they showed that immune responses were mediated by the membrane permeability and assessed that, using muscle-specific promoters, the activation of the immune system was significantly delayed [80]. Starting from data from DMD clinical trials that confirmed how the therapy with prednisone ameliorated the pathological phenotype and strength of muscle [81], they suggested that immunosuppression not only ameliorated the efficiency of transduction mediated by the AAVs but above all limited the degeneration of myofibers blocking the immunological cells that recognize and destroy newly formed dystrophin positive myofibers [80]. More recently, Mendell and coworkers treated a group of six DMD patients with AAV carrying a minidystrophin transgene that partially restores the generation of muscle force in dystrophic mice [15]. As expected, following the treatment, they identified dystrophin-specific T cells but, surprisingly, circulating dystrophin-specific T cells were found in two patients before AAV treatment. Furthermore, they assessed that autoreactive T cells recognized epitopes that were presented on revertant dystrophin fibers. This way, they suggested that to increase the efficiency of experimental therapy for DMD in term of formation of dystrophin positive myofibers, T-cell immunity to self and nonself dystrophin epitopes has to be accurately investigated [15]. Recently, Flanigan et al. evaluated dystrophin-specific T cell immunity in DMD patients that were treated with glucocorticoid steroids [82]. They showed that not only the risk for the presence of antidystrophin T cell immunity increased with age but, more interestingly, steroid-treated patients developed milder immune response related to not treated patients. Steroids could exert their beneficial effects in DMD patients by modulating T cell responses [82]. In addition to AAVs, HIV-derived lentiviral vectors were used to transfer genes to muscle; they can infect both dividing and nondividing cells and possess the capacity to clone minidystrophin together with selectable markers and the promoters needed for transgene expression [83]. These vectors permanently transduce and stably express transgenes in muscle cells (and their precursors) [84, 85]. Unfortunately, their use in clinical approaches was largely restrained for safety reasons. HIV could self-replicate and produced during manufacture of the vector in the packaging cell line or in the target cells by a process of recombination; consequently, the patients undergoing gene therapy would be infected with HIV in addition to the new therapeutic gene. Moreover, self-replicating infectious vector could cause uncontrolled insertion into host genome and activate prooncogenes causing cancer [86].

#### 4.2.2. Gene Therapy Applicability in LGMD and FSHD

Differently from dystrophin, the full-length cDNA of each isoform of the sarcoglycans (whose mutations cause the LGMD) can be cloned into the AAV so that AAV-based gene therapy is feasible for this disease. In different studies, Li and Greelish demonstrated the feasibility of AAV-mediated gene transfer of *δ*-sarcoglycan into skeletal muscle using cardiomyopathic hamster [87, 88] but this model—as the majority of the murine ones—did not face the pathological condition of LGMD muscles. In fact, the rate of ongoing degeneration-regeneration cycles is too slow so that muscle maintains its function and fibrosis did not develop well. This way, Hack et al. developed a new murine model for primary *γ*-sarcoglycan deficiency that exhibited the clinical and histopathological characteristics of LGMD: degeneration and regeneration of muscles, pseudohypertrophy, and development of fibrosis. All of these features became evident as early as 4 weeks, following muscle membrane disruption [89]. Cordier et al. used this model to test the efficacy of AAV-mediated transfer of *γ*-sarcoglycan; moreover, to avoid possible toxicity and immunogenicity in nontarget tissues, they used muscle-specific promoter to express the gene only into differentiated muscle [90]. They found that delivery of AAV allowed long-term correction of the disease phenotype and, more interestingly, fibrosis presented a significant barrier for viral delivery. As fibrosis is the last of a series of events that are initiated by the recruitment of immune cells and APCs into muscle to counteract the presence of AAV and its transgene, they suggested that gene therapy has to be performed early in the life of the patients, before that fibrosis occurs [90]. It is now known that FSHD is caused by gene overexpression [91]. The group of Gabellini developed a murine model of FSHD overexpressing the FRG-1 gene that resembled histological and molecular features typical of the disease [3]. Due to its genetic features, FSHD provides a valuable model to test therapeutic potentials of RNAi-mediated gene silencing. Bortolanza et al. combined the delivery mediated by rAAV6 with RNAi-mediated mRNA knockdown and injected rAAV6 expressing FRG1 shRNAs into the tail vein of FRG1 animals [92]. They demonstrated that the treatment was safe and allowed the long-term knockdown of FRG1. Furthermore, the rAAV6 injection ameliorated the pathological phenotype of mice; in particular, the complete functional recovery of the muscular functions was obtained only with the higher dose of vectors. In this contest they did not assess any problem of immune reaction against the product of the vector but they observed that the expression of RNAi cassette could be toxic [92]. As this effect was observed in other diseases [93, 94], it was determined that toxicity was abolished as soon as RNAi hairpin sequences were captured by naturally occurring miRNA scaffold [93].

## 5. Cell Therapy and Immune Responses

Cell therapies have gained increased attention in the last years. Therapeutic cells could be obtained from patient, corrected* ex vivo,* and retransplanted (autologous implantation). Alternatively, the cells could be isolated from healthy donors and injected into dystrophic patient (allogeneic implantation). The most suitable cells for therapeutic purposes should be easily isolated and retain the capacity to migrate from blood to muscle and to enter the satellite cells niche. Once the host cells fuse, affected muscles will repopulate improving muscle function and pathology. Stem cells were showed both to replenish their numbers for long periods through cell division and to efficiently produce a progeny, differentiating into multiple cell lineages [95]. Embryonic and adult stem cells differ significantly with regard to their differentiation potential and* in vitro* expansion capability. Adult stem cells constitute a reservoir for tissue regeneration throughout the adult life; they are tissue-specific and possess limited capacity to be expanded* ex vivo*. Conversely, embryonic stem cells (ESC) are pluripotent cells derived from the early embryo; they are capable to proliferate over prolonged periods of culture, to remain undifferentiated, and to maintain a stable karyotype [96–98]. In the next section, we will examine the studies in which these cells are involved.

### 5.1. Immunogenicity of Embryonic Stem Cells and Induced Pluripotent Stem Cells

Different works described good results of engraftment following the injection of ESCs into recipient muscle [99, 100]. Although ESCs could represent reliable and cost-effective therapeutic substitute for treatment of neuromuscular disorders, their transplantation often causes teratomas so that all undifferentiated cells need to be removed from a graft. Taken together with ethical problems in the destruction of the blastocyst, the employment of ESCs in a clinical perspective is far from occurring. In 2006, the group of Yamanaka obtained ES-like induced pluripotent stem cells (iPSCs) from adult mouse and human cells by introducing specific sets of genes encoding for transcription factors expressed in undifferentiated ES cells to reprogram the adult cells [101]. Similar to ESCs, iPSCs retained the ability to differentiate into all adult cell types and, more importantly, their generation does not imply the use of embryonic or foetal material. However the safety of these cells had to be tested accurately before attempting any therapies [101]. Since ESCs were discovered, they were thought to be immune-privileged as their low expression of MHC class I, MHC class II and, conversely, high expression of immunomodulatory molecules regulating the proliferation of T-cell [102]. Unfortunately, it was demonstrated that ESCs allowed donor-specific immune response in immunocompetent mice [103]. The findings of iPSCs opened new possibilities to solve the problem of immune rejection but some hurdles remained. Very preliminary study described that iPSCs were rejected even in MHC-matched recipients, due to unnatural expression of genes that were recognized by CD4+ and CD8+ T cells [104]. Mullally and Ritz suggested that genomic alterations acquired during iPSCs formation/proliferation generated immunogenic neoantigens, potentially eliciting immune responses even in a MHC-matched context [105]. Their observations were confirmed by another work, showing that iPSCs had uncontrolled differentiation capacity due to duplications on chromosome 12 [106]. Furthermore, several studies demonstrated that ESCs rejection was accelerated during upregulation of MHC [103, 107, 108] so that it was suggested that ESCs/iPSCs transplantation could be more difficult into such an environment. Other studies focused on minor histocompatibility antigens (miHA) and determined that even identical HLA phenotype could not be sufficient to guarantee graft survival [109, 110]; in addition, expression of Oct-4 and other specific factors could enhance miHA incompatibilities [111]. It is demonstrated that immunonological problems following cellular injection can be at least diminished by eliminating APCs from the graft before transplantation [112]. As endothelial cells are able to mediate the direct pathway of allorecognition [113], the use of ESCs and iPSCs derived from endothelial cells needs further precautions.

### 5.2. Immunogenicity of Myogenic Stem Cells

Initial efforts were focused on the progenitor cell in skeletal muscle, the satellite cell, and the descendants of activated satellite cells, the myoblasts. Partridge's group demonstrated that transplantation of wild-type syngeneic myoblasts restored dystrophin expression in immunodeficient mdx mice [114]. Although the first DMD trials based on myoblast transplantation showed a partial expression of dystrophin [76, 78, 79], they failed for donor myoblast survival, as they undergo rapid and massive death after injection into host muscle. It was suggested that host immune response was responsible for the death of the transplanted cells. In particular, taking into account the rapidity with which donor myoblasts die following transplantation, it was thought that the complement system allowed the generation of a membrane attack complex (MAC) through the expression of a C3 convertase, lysing targeted cells quickly. In case of complement depletion, the survival of donor myoblasts was not enhanced so that it was argued that complement could induce the activity of neutrophils and macrophages, indirectly regulating the death of transplanted cells [115]. Different studies demonstrated that myoblast could be recognized directly by host T cells due to the expression of MHC antigens that are recognized by TCR on T cells. Alternatively, myoblasts could be identified by APCs that present donor antigens to host T-cells [116, 117]. Furthermore, it was supposed that muscle-resident mast cells that are elevated in DMD patients [118] secreted cytokines such as TNF-*α*, affecting donor myoblast survival [119]. Data obtained by these studies lead to the conclusion that immune suppression was necessary to permit allogeneic myoblasts transplantation [76]. Several studies were conducted using different immunosuppressive protocols, in attempt to avoid side effects of systemic immunosuppression while permitting donor myoblasts survival. Unfortunately none of these studies reported significant or long-term improvement in muscle strength [120–123]. It was thought that stem cells, differently from myoblasts, were immune-privileged so that the risk for immunorejection was underestimated. Today the immunogenicity of stem cells has been widely demonstrated both for embryonic and adult cells [111, 124, 125]. In particular autologous stem cells can provoke inflammation and rejection maybe as a consequence of genetic manipulation or long-term culture (required for their correction and expansion) or their combination with matrix structures. Similarly, ESCs express low levels of HLA class I but only before differentiation; once injected and after reaching the host organ, the MHC disparities lead to acute antibody-mediated rejection (AMR) [126]. Furthermore if IgG antibodies directed against HLA class I antigen of donor cells are already present at the time of transplantation hyperacute rejection (HAR) will occur [127, 128]. Interestingly evidences arose that HLA/MHC molecules could have a role in signalling transduction so that the prediction of efficacy of transplantation is further reduced. MHC expression in transplanted cells could be influenced by the host microenvironment (e.g., INF-*γ* or hypoxia exposure) [126]. Anti-HLA antibodies mediated injury through both complement-dependent and independent pathways and through the binding with HLA donor cells; this binding could result in activation, proliferation, and cytokines production, leading to amplification of damage [129]. Interestingly, HLA signalling is not a prerogative of immune cells alone, as it also occurs in endothelial or epithelial cells with unknown side effects [130, 131]. Other than satellite cells and myoblast, muscular and nonmuscular multilineage stem cells able to actively participate in myogenesis were identified and characterized according to the expression of different cellular markers [132–138]. However, promising results obtained with animal models were not replicated in humans. In addition, only some of those cells migrated through the vasculature (CD133+, mesoangioblasts, and mesenchymal stem cells) [133, 137, 139, 140] and, this way, were considered for therapeutic interventions. In 2009, Cossu's group started a clinical study in DMD patients ranging in age from 5 to 12 years. The ongoing clinical trial considered intra-arterial infusion of donor mesoangioblasts HLA-identical donor derived in DMD patients pretreated with Tacrolimus. Results of the immune-reaction in this study are in progress.

### 5.3. Immunomodulation Behaviour of Mesenchymal Stem Cells

Mesenchymal stem cells (MSCs) are currently considered as one of the most promising cell types for cell therapy. Firstly isolated from bone marrow [141], and nowadays obtained from a variety of tissues [142, 143], they are a heterogeneous cell population characterized through different culture conditions and surface markers expression. Apart from the multipotency of cell differentiation, MSCs have been shown to modulate endogenous tissue by secreting a large spectrum of bioactive molecules [144] that were demonstrated to induce different responses, such as angiogenesis [145], inflammatory inhibition, immune modulation, and apoptosis reduction [146]. Furthermore, it is well known that MSCs are immunoprivileged due to their low expression of MHC-II and costimulatory molecules in their cell surface, making them invisible to immune system. The mechanism of action underlying this behaviour involves different immune cell types, both from innate and adaptive immune response. As regards the first type of response, for example, MSCs are able to prevent the two phases of DCs maturation—from precursors to immature state (iDC) [62] and the complete maturation [147, 148]. In both cases MSCs generate a tolerogenic fate of DCs and a possible switch from Th1 to Th2 response [149]. On the other hand, as regards the natural killer cells (NKs), MSCs can prevent their activation and consequent massive release of IFN-*γ* and TNF-*α*, with resulting cytotoxic effect [150, 151]. Only if NKs are not previously activated by IL-15 or IL-2, MSCs act as block for NKs lytic ability [152]; otherwise, NKs are able to kill allogeneic and autogenic MSCs, in a process mediated by specific molecules [150, 152]. In the prospective of an application of MSCs in cell therapy, it has to be taken into consideration the possibility that the activation of NKs, due to infections or tumor cells, could interfere to the therapeutic effects of transplanted MSCs. On the contrary, the presence of important concentrations of MSCs could also turn off the innate immune responses of NKs against a future infection or neoplastic development [153]. As regards the adaptive immune response, aspects such as proliferation, differentiation, and maturation of B-cells and their antibody production can be affected by the presence of MSCs in a dose-dependent way [154–157]. MSCs immunosuppressive mechanism is based principally on B lymphocyte-induced maturation protein-1 (Blimp*-*1) blocking, whose expression is necessary for Ig production [154, 156]. Also T-cell responses are affected by MSCs properties; proliferation, release of IFN-*γ*, and cytotoxicity are influenced in a dose-dependent manner [158]. All these effects seem to be mediated by the release of soluble factors, such as TGF-*β* and IL-10; in addition, the nitric oxide (NO) should play a major role in the MSC-mediated T-cell suppression, through a mechanism of inhibition of signal transducer and activator of transcription 5 (STAT5) phosphorylation that prevents T-cells from entering the cell cycle [159–161]. In both cases (regulation of B- or T-cells), the MSCs need to be previously activated to exert their suppressive properties. In a synergic view of immunosuppressive behaviour, MSCs increase the proliferation of T-regs, immune cells capable of suppressing the proliferation of activated T cells, in order to avoid a host damage due to an exaggerated immune response. In fact, it has been demonstrated* in vitro* that when MSCs are added to a mixed lymphocyte reaction the percentage of T-reg increases [147], while* in vivo*, the same result is reached after the injection of MSCs intravenously [162]. Several studies demonstrated the ability of MSCs to diffuse in dystrophic host muscles once injected in utero- or intravenously in mdx mice together with some dystrophin reexpression [163–165]. Although these studies confirmed the possibility of MSCs transplantation without immunosuppression, they did not report any increase in muscle strength [166, 167]. Recently Vieira et al. demonstrated that human adipose-derived mesenchymal stromal cells were able, once injected systemically into GRMD dogs, to reach the host muscle and to express human dystrophin without any need of immunosuppression [168]. These data are the starting point for the development of new therapeutic strategies.

## 6. Conclusions

In MDs the chronic progression of the disease leads to exhaustion of muscle regenerative potential, so that gene therapy will result to be useless without supplying new muscular fibres. In this point of view cell therapy represents the best tool to deliver both the functional gene and the myogenic potential required. Gene therapy is focused on replacing the defective gene with a new one carried by different vectors; AAVs-mediated gene therapy for MDs is a feasible therapeutic approach that leads to the planning and implementation of phase I clinical trials. Initially on preclinical studies, the treatment with AAVs seemed to be minimally immunogenic, but data from limited human trials evidenced the concept of vector dose-dependent immunotoxicity [169, 170]. Moreover, transgene cassette and capsid structure have been shown to stimulate an immune response [171, 172]. As it became evident that unique AAV serotype will not be universally applicable for therapeutic gene transfer, other naturally occurring alternate AAV serotypes have to be developed and utilized [173, 174]. Moreover, modifications of these vectors could increase their transduction efficiency and consequently reduce dose-dependent immune response. To guarantee a relevant restoration of muscle function in DMD patients, it is necessary to perform repetitive AAV infusions to target multiple muscular territories. Many works showed the decrease of the efficiency of gene transfer after repeated injections of the AAV2 capsid in normal mice. Unfortunately, there is no animal model which can accurately predict the anti-AAV immune response in humans [59]. MDs, despite their heterogeneity, share some common features like progressive muscular weakness, atrophy, and inflammation. Furthermore, chronic injury determines* per se* immune system activation. The immune system response has different components: the humoral immunity, the cell mediated immunity, and the inflammatory pathway that includes the complement system and the macrophages or NKs activation. All these components are involved at different levels in each MDs form and they are differently activated against cell- or gene-therapy both in a specific manner, linked to the type of cell or vector used, and according to MDs-specific immune-pathogenetic mechanisms.

A new emerging point is the regulation of genes involved in immune response which are overrepresented in human population, so that some pathways are subjected to positive or balancing selection [175, 176]; in particular, they were identified genes related to cytokines (IL-1 receptor agonists) and MHCI-related antigen presentation (as TAP1) [177–179]. These genetic variants are responsible for phenotypic diversity and adaptation against viral/bacterial infection but also to susceptibility to autoimmune diseases. The genetic predisposition could be involved both in variability of response to treatment (as differences among DMD patients in steroid's treatment response) and in immunity response against gene therapy. In fact the ability to present antigen with MHC-I molecules could influence the number of antidystrophin CD4+ T cells produced after AAV injection in DMD patients. In the future, patients could be characterized for their genetic predisposition to develop an immune response as a tool for patient risk stratification and to administrate high-dose of immunosuppression only if it is indispensable.

*Humoral Immunity in MDs.* Partial benefits were observed in LGMD2B patients with intravenous immunoglobulins and Rituximab (anti-CD20) administration; both of these treatments blocked B-lymphocytes which are not a major component of infiltrates in this disease [43], suggesting that further studies are needed to clarify the role of B-lymphocytes in this muscle disease. Among alternative strategies to bypass the problem of immune response, the immunomodulatory effect of intravenous immunoglobulin (IVIG) was used in immune-mediated diseases such as multiple sclerosis and myasthenia gravis [180, 181] and, largely, in neuromuscular diseases [182]. It was postulated that the IVIG in association with cell and gene therapy could modulate the immune system via multiple putative mechanisms. Therefore, as a theoretical rationale for the use of IVIG treatment was demonstrated for inflammatory diseases [183], the specific effect in MDs has to be completely understood. Lorain et al. described the humoral immune response AAVs following intramuscular injection of AAV1-U7. As it could preclude the success of subsequent AAV1 infusions if administered more than 3 days after the primary injection, they eliminated this adverse immunity by means of CTLA4/Fc and MR1, which are currently being evaluated in human clinical trials [67]. CTLA4/Fc has been used successfully in animal models of autoimmunity [184] and transplantation [185] and has been approved by the Food and Drug Administration for clinical use. Other studies showed that AAV vectors may induce humoral adaptive response to AAV* in vivo* as the vectors interact with different complement components [186]. Therefore, these results indicated that it is necessary to combine gene therapy with immunosuppressive or immune-modulant therapy to prevent immune system activation and to allow the expression of the transgene.

*Cell Mediate Immunity in MDs*. In DMD a self-directed T cell activation is emerging as the major obstacle to gene or cell therapy as T-CD4+ cells directed against dystrophin protein and not against exogenous vector or donor cells were observed [15]. This evidence suggests that immunosuppressive therapy should be coupled with other preventive strategies to induce persistent antigen-specific tolerance in the gene therapy setting [187]. This cell-mediated autoimmune component was never observed in dysferlinopathies, where altered macrophages function, complement deposition, and cytokines release are the major features [22]. Only poor data are available on FSHD pathophysiology so that specific immune mechanism is not yet described. Steroid treatment is the only therapeutic strategy available for DMD. Interestingly Flanigan et al. demonstrated that steroids also reduced the number of patients' autoreactive antidystrophin T-cells [82]; accordingly, steroid treatment could be used as immunomodulator together or before starting a gene or cell therapy to facilitate the survivor of dystrophin expressing fibers. Unfortunately both in dysferlinopathies and in FSHD, steroids' clinical trials failed to ameliorate or delay the disease progression [30]. In case of DMD, cell therapy focused on administration of dystrophin-expressing myoblasts. Tremblay et al. transplanted myoblasts in 5 patients without cyclosporine. Unfortunately, no increase in isometric force was observed and, 6 months after the injection, less than 1.5% of dystrophin-positive fibers were found. This study demonstrated that immune suppression was necessary for the transplantation of allogeneic myoblast [76]. However, in 1993, Karpati et al. showed that no functional improvement or dystrophin expression were found after transplantation of 55 million of myoblasts in the biceps of 8 DMD patients under cyclophosphamide immunosuppressive treatment [120]. Since the problem arose with myoblast transplantation, many research groups focused their attention on stem cells but the risk for immunorejection was underestimated. In fact ESCs once differentiated in the host tissues' ability to lose their “immune-privilege state” thanks to MHC class I expression, favoured by host microenvironment [126]. Similarly IPSCs can be recognized by CD4+ and CD8+ T-cells through unnatural-manipulated genes and also autologous stem cells can be modified by long-term culture or supports materials [111, 124, 125]. As we stated before, the immunomodulatory properties of MSCs are so appealing to consider these cells for clinical purposes. Many studies demonstrated that the therapeutic effects of MSC are due not only to direct differentiation into injured tissue but also to production of paracrine factors that are able to inhibit apoptosis, to increase endogenous cell proliferation, and/or to stimulate tissue resident stem cells in the site of injury [188]. Together with MSCs, several stem cell populations (resident and nonresident in muscle) were investigated for their ability to ameliorate the pathological phenotypes of muscular dystrophies. Among them, some not only were differentiated into muscle but, more importantly for the treatment of devastating muscle disease, retained the ability to migrate through vasculature and reach all the muscles of the body. Our group demonstrated that CD133+ stem cells isolated from muscle can be injected safely into DMD patients [189] and, genetically modified, improved muscle function and allowed dystrophin expression following transplantation into dystrophic mice [133]. According to these results, we will start a clinical trial where we treat DMD patients with their own CD133+ stem cells* ex vivo* engineered with a lentiviral vector carrying the AONs sequences able to skip the exon 51. Similarly, very promising results were obtained by injecting muscle-derived stem cells (MDSCs) [190, 191] and mesoangioblasts [192–194] into dystrophic animal models. An ongoing clinical trial, promoted by Cossu's group, will assess the feasibility of intra-arterial transplantation of mesoangioblasts, from HLA-identical donors, in DMD children treated with bland immune suppression.

In the last years, many ways were undertaken in order to find the best conditions to decrease the host immune reaction after gene or cell therapy. In 2013, Figueiredo et al. demonstrated that MHC class I silencing significantly prolongs cell survival after allogeneic transplantation by preventing the identification from the immune system. Silencing MHC expression on transplanted cells could represent a potential field able to revolutionize the cell-based products developed for regenerative medicine and particularly for muscular dystrophies treatment [195].

*Inflammatory Pathway in MDs.* During tissue regeneration events, infiltrating inflammatory cells and resident cells interacts precisely. Impairment of these events can cause unsuccessful regeneration and develop a condition of injury, typical of the MDs (as described above in the Section 2 “Immune system activation in skeletal muscle”). To defeat inflammation is one of the most important goals of clinical experimentations, as transplantation of cells into such an environment limited dramatically their survival, due to activation of macrophages.

Steroids are efficient not only in modulating cell-mediated immunity but also in attenuating the inflammatory pathways involved in MDs. As they are associated with important adverse effects, it could be important to better elucidate the factors that drive inflammation to obtain more selective immunomodulatory intervention. As discussed above, impairment in dysferlin-mediated membrane repair promotes a destructive inflammatory response by activating the innate immune system. However, all the pharmacological treatments tested failed to work in dysferlin-deficient patients [41, 42]. To date, encouraging results were obtained by studying the NLRP3 inflammasome signalling pathway so that it is now considered as a good therapeutic target for dysferlinopathy [196]. As DMD muscle continuously express chemokines, it is thought that muscle itself contributes to the chemotaxic process, causing the chronic inflammation; this way, the pathways activated by these molecules are considered interesting candidates for immunosuppressive therapies. Other treatments using new immunomodulatory drugs such as chemokine-receptor antibodies or cytokines neutralizing antibodies (Infliximab and Etanercept) are still confined to preclinical studies, although they seem promising in dystrophic animal models. In addition, it is known that MCSc regulate biological processes associated with inflammation and suppress various immune functions, through release of immune suppressive cytokines and production of soluble HLA-G [188]. As we discussed above M1/M2 balance influences the inflammatory environment; we previously demonstrated an amelioration of dystrophic features in scidA/AJ mice consequent to T and B depletion and M2 macrophages switch [17]. Similarly, the group of Tidball demonstrated that anti-inflammatory IL-10 reduced the pathology of mdx muscular dystrophy by deactivating M1 macrophages [12, 197]. Conversely, an inflammatory environment could be beneficial in cell therapy as injected cells could be attracted to the site of injury and they could be facilitated in the chemotaxic process by chemokines/cytokines.

In conclusion, the ability to regulate the expression level of a therapeutic gene and to control the immune response is vital to proceed with gene therapy in clinic. Gene replacement strategies offer the potential for long-term correction. Improved gene therapy vectors together with advances in bypassing immune responses provide a platform for meaningful translation to patients. In the field of muscular dystrophies the combination of cell and gene therapy is the most promising as it is important to overcome the regenerative potential exhaustion. Engineered stem cells could provide new muscular fibers and decrease the immune response of the host; particularly, the application of myogenic stem cells as a possible cell or gene-cell combined therapy represents a very interesting tool, especially in tissues that are characterized by a chronic inflammation. However, we need to better clarify the immunopathogenetic mechanism underlying the different forms of MDs in order to develop more selective immunotherapies combined with cell and gene therapy.
